# New deep-sea Amphipoda from the Clarion-Clipperton Zone: 24 new species described under the Sustainable Seabed Knowledge Initiative: One Thousand Reasons campaign

**DOI:** 10.3897/zookeys.1274.176711

**Published:** 2026-03-24

**Authors:** Anna M. Jażdżewska, Tammy Horton

**Affiliations:** 1 Department of Invertebrate Zoology and Hydrobiology, Faculty of Biology and Environmental Protection, University of Lodz, Lodz, Poland National Oceanography Centre Southampton United Kingdom; 2 National Oceanography Centre, Southampton, UK University of Lodz Lodz Poland

**Keywords:** Amphipod crustaceans, manganese nodules, new species, Pacific Ocean

## Abstract

The Clarion-Clipperton Zone (CCZ) in the central east Pacific holds significant abundances of manganese nodule deposits. The region is subject to intense exploration for potential future deep-sea mining. One of the challenges associated with understanding potential impacts of extraction of these nodules is the insufficient knowledge of the CCZ fauna. In response to the need for improved taxonomic knowledge of the CCZ fauna, the International Seabed Authority (ISA) initiated the Sustainable Seabed Knowledge Initiative: One Thousand Reasons campaign, aiming to describe 1000 species new to science before the end of the decade. Within this call, a taxonomic workshop “New species of Amphipoda from the Clarion-Clipperton Zone” took place at the University of Lodz, Poland in February 2024, providing the impetus for the description of 24 new species of amphipods from the central Pacific abyss. This special issue is devoted to the publication of the new species from the workshop, and we summarise those findings here.

## Introduction

This publication is not deemed to be valid for taxonomic/nomenclatural purposes (see Article 8.2 in the International Code of Zoological Nomenclature 4^th^ Edition [International Commission on Zoological Nomenclature 1999]).

The Clarion-Clipperton Zone (CCZ) situated between the Clarion and the Clipperton Fracture Zones in the central east Pacific and covering approximately 6 million km^2^ holds major portions of manganese nodule deposits and is therefore subject to intense exploration for future deep-sea mining activities (Hein et al. 2020). One of the challenges associated with proper evaluation of deep-sea mining impact is insufficient knowledge of the abyssal faunal diversity and their assemblages (e.g. Washburn et al. 2021; Uhlenkott et al. 2023).

A recent summary of the known diversity of metazoans across the CCZ region revealed 5,578 species recorded in the CCZ region of which between 88 and 92% were classified as unnamed species (Rabone et al. 2023). Description of new species is rarely funded, and as a result many taxa identified as part of baseline surveys, and recognisable as distinct entities (including morphologically identified Operational Taxonomical Units [OTU] and Molecular Operational Taxonomic Units [MOTU]), remain delineated only by temporary names or codes (Glover et al. 2018; Horton et al. 2025). Such an approach allows for preliminary assessment of biodiversity, but unless these temporary names are very well-defined and published, they are not recognised by the scientific community or society, and only formally described species can become a subject of conservation (Delić et al. 2017; Britz et al. 2020; Horton et al. 2025). Moreover, only morphologically characterised species can be compared with historical collections or recent material unavailable for molecular studies (Dupérré 2020). The description of new species provides a baseline for further ecological or biogeographical studies and as such it is a crucial and the only sustainable step in recognition of species and their service for ecosystem functioning.

### The Sustainable Seabed Knowledge Initiative: One Thousand Reasons campaign

The original concept for the “One Thousand Reasons” campaign was mooted in a publication authored by taxonomists in 2018. In this publication, the lack of descriptive taxonomy of the CCZ fauna, despite decades of research, hundreds of expeditions and many thousands of samples having been collected, was decried (Glover et al. 2018). Glover et al. argued that the reason for the “historic failure of taxonomy on the high seas” was a lack of resources for post-expedition analyses and archiving of samples and data. The authors then suggested a potential solution to what was generally seen as an insurmountable problem. They recommended that a reasonable solution would be for the regulator, the International Seabed Authority (ISA), to set targets to describe 100 species per year over a decade, as well as ensuring accessible, vouchered material and genetic data (Glover et al. 2018). The ISA jointly launched the Sustainable Seabed Knowledge Initiative (SSKI) with a range of partners in June 2022, and in May 2023 launched the first “One Thousand Reasons” campaign to support taxonomic research projects aimed at describing new deep-sea species (https://www.isa.org.jm/sski/).

Very few comprehensive studies of Amphipoda from the CCZ have been conducted to date, and those that have been carried out have largely focussed on the mobile scavenging fauna attracted to baited traps (Patel et al. 2020; Bribiesca-Contreras et al. 2021; Jażdżewska et al. 2021; Jones et al. 2021; Mohrbeck et al. 2021; Stewart et al. 2024). The few works on amphipods found within boxcores or epibenthic sledge nets have been taxonomic in focus (Jażdżewska et al. 2022) or have remained as unpublished reports or theses.

The current CCZ species list comprises just 13 described species of amphipods from seven genera in seven families (Rabone et al. 2025; https://www.marinespecies.org/deepsea/CCZ/index.php). Eleven of these species are scavenging amphipods which have also been reported more widely from other regions and are in some cases considered cosmopolitan. Only two species of non-scavenging amphipod have been described from the CCZ region to date (*Oedicerina henrici* Jażdżewska in Jażdżewska, Brandt, Martínez Arbizu and Vink 2021 and *O. teresae* Jażdżewska in Jażdżewska, Brandt, Martínez Arbizu and Vink 2021; Jażdżewska et al. 2021). Although there are very low numbers of named amphipod species reported from the CCZ, the number of OTUs and MOTUs is much larger—comprising of at least 28 families, 41 genera, and 16 species-level taxa according to Rabone et al. (2023), and >200 MOTUs according to Jażdżewska et al. (2025).

Tackling this disparity between the number of named versus unnamed amphipod taxa in the CCZ was the primary aim of the workshop funded through the SSKI program. In February 2024, a group of eight taxonomic experts and eight students and early-career researchers gathered at the Department of Invertebrate Zoology and Hydrobiology, University of Lodz for a taxonomic workshop devoted to describing new species from the CCZ. This workshop resulted in the preparation of 14 manuscripts describing one new family, two new genera and 24 species new to science from 11 amphipod families, which are presented in this Special Issue.

## Methods

**Figure 1.**
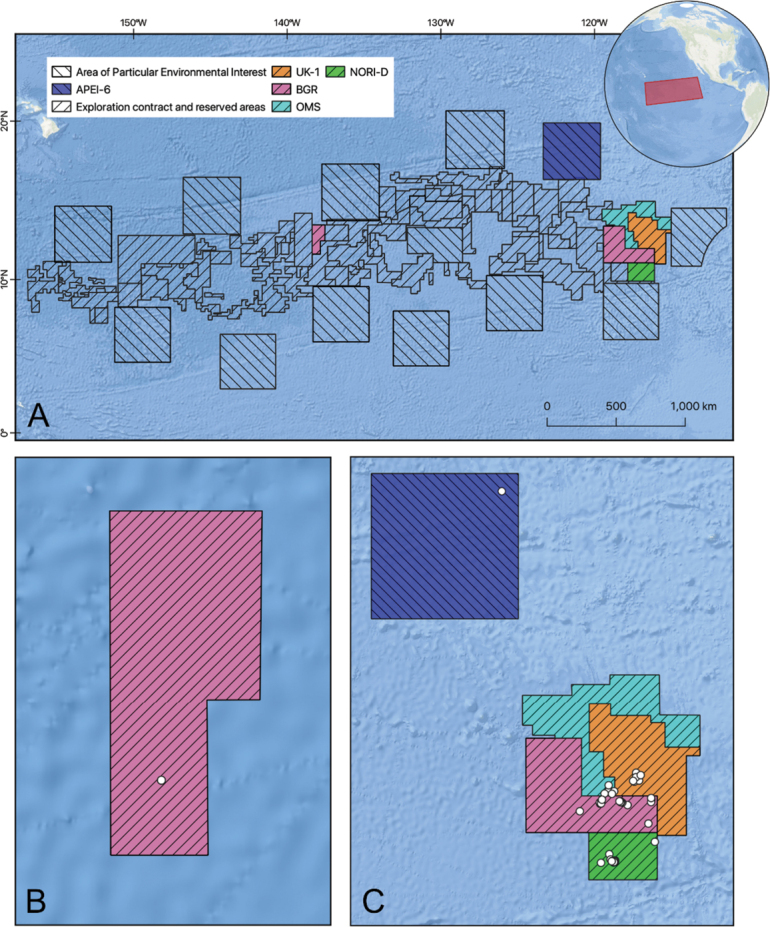
Map of the Clarion-Clipperton Zone (CCZ), Central Pacific Ocean. **A**. Exploration contract areas from which samples were collected; inset map shows the general location of the CCZ; **B**. Detail of the central BGR exploration contract area; **C**. Detail of the eastern CCZ exploration contract areas and APEI 6. Points indicate epibenthic sledge, boxcore, and multi-corer sample locations taken across all expeditions.

The material for all papers presented in this Special Issue was sampled from the central-east Pacific Ocean, with the majority being collected in the easternmost sector of the Clarion-Clipperton Zone (CCZ) and just a single sample from the central BGR exploration contract area (Fig. 1, Table 1).

**Table 1.** Station data including sampling area (UKSR-1: UK Seabed Resources Ltd, United Kingdom, OMS: Ocean Metals Singapore, BGR: Bundesanstalt für Geowissenschaften und Rohstoffe, NORI-D: Nauru Ocean Resources Inc., APEI 6: Area of Particular Environmental Interest) and sampling gear information (EBS – epibenthic sledge, BC – box corer, MC – multi corer).

Expedition	Station	Area	GPS Coordinates	Depth [m]	Collection date	Gear
ABYSSLINE-2	AB2-EB01	UKSR-1	12°22.02'N, 116°33'W	4209	18-Feb-2015	EBS
ABYSSLINE-2	AB2-EB02	UKSR-1	12°31.86'N, 116°37.38'W	4219	20-Feb-2015	EBS
ABYSSLINE-2	AB2-EB03	UKSR-1	12°33.78'N, 116°37.5'W	4219	23-Feb-2015	EBS
ABYSSLINE-2	AB2-EB04	OMS	12°7.8'N, 117°18.66'W	4111	25-Feb-2015	EBS
ABYSSLINE-2	AB2-EB05	OMS	12°6.12'N, 117°10.86'W	4100	26-Feb-2015	EBS
ABYSSLINE-2	AB2-EB06	OMS	12°15.06'N, 117°19.2'W	4137	01-Mar-2015	EBS
ABYSSLINE-2	AB2-EB07	UKSR-1	12°27.06'N, 116°37.8'W	4145	02-Mar-2015	EBS
ABYSSLINE-2	AB2-EB08	UKSR-1	12°30.18'N, 116°30.54'W	4244	04-Mar-2015	EBS
ABYSSLINE-2	AB2-EB09	UKSR-1	12°21.6'N, 116°42'W	4170	10-Mar-2015	EBS
ABYSSLINE-2	AB2-EB11	OMS	12°2.28'N, 117°14.22'W	4097	14-Mar-2015	EBS
ABYSSLINE-2	AB2-EB12	OMS	12°2.7'N, 117°25.44'W	4223	16-Mar-2015	EBS
ABYSSLINE-2	AB2-EB13	APEI 6	19°27.9'N, 120°1.5'W	4026	20-Mar-2015	EBS
MANGAN 2016	Ma 16-18	BGR	11°51.372'N – 11°51.662'N, 117°01.535'W – 117°00.482'W	4132-4123	28-Apr-2016	EBS
MANGAN 2016	Ma 16-25	BGR	11°49.143'N – 11°49.975'N, 116°58.492'W – 116°57.797'W	4107-4101	29-Apr-2016	EBS
MANGAN 2016	Ma 16-28	BGR	11°49.654'N – 11°49.902'N, 117°00.299'W – 116°59.174'W	4143-4133	01-May-2016	EBS
MANGAN 2016	Ma 16-91	BGR	11°49.792'N – 11°49.842'N, 117°30.458'W – 117°29.208'W	4344-4344	09-May-2016	EBS
MANGAN 2016	Ma 16-95	BGR	11°47.862'N – 11°47.152'N, 117°30.639'W – 117°29.490'W	4356-4359	09-May-2016	EBS
MANGAN 2016	Ma 16-96	BGR	11°53.492'N – 11°53.791'N, 117°29.357'W – 117°28.209'W	4193-4256	10-May-2016	EBS
MANGAN 2018	SO 262-59	BGR	11°49.720'N – 11°50.055'N, 117°01.080'W – 116°59.530'W	4097-4128	22-Apr-2018	EBS
MANGAN 2018	SO 262-67	BGR	11°51.190'N – 11°51.621'N, 117°02.830'W – 117°00.804'W	4131-4131	24-Apr-2018	EBS
MANGAN 2018	SO 262-150	BGR	11°50.009'N – 11°49.978'N, 116°14.780'W – 116°13.316'W	4074-4095	07-May-2018	EBS
MANGAN 2018	SO 262-151	BGR	11°55.986'N – 11°55.992'N, 116°14.706'W – 116°13.320'W	4150-4151	08-May-2018	EBS
MANGAN 2018	SO 262-155	BGR	11°47.436'N – 11°47.677'N, 117°32.213'W – 117°30.910'W	4352-4351	09-May-2018	EBS
MANGAN 2018	SO 262-156	BGR	11°49.381'N – 11°49.752'N, 117°32.663'W – 117°30.760'W	4340-4340	09-May-2018	EBS
MANGAN 2023	KM23-7	BGR	12°02.2976'N – 12°03.5888'N, 138°05.4932'W – 138°05.7092'W	5109-5139	17-Apr-2023	EBS
MANGAN 2023	KM23-50	BGR	11°17.7919'N – 11°18.5445'N, 116°18.8626'W – 116°17.6747'W	4185-4182	01-May-2023	EBS
MANGAN 2023	KM23-69	BGR	11°36.252'N – 11°37.1050'N, 118°02.981'W – 118°01.2511'W	4368-4356	04-May-2023	EBS
MANGAN 2023	KM23-74	BGR	11°47.6444'N – 11°48.0414'N, 117°30.9650'W – 117°29.5413'W	4360-4364	06-May-2023	EBS
MANGAN 2023	KM23-79	BGR	11°51.3560'N – 11°51.7516'N, 117°01.2662'W – 116°59.8924'W	4126-4128	07-May-2023	EBS
Resource Cruise 01 (RC01)	BC_028	UKSR-1	12.3390°N, 116.6690°W	4158	09-Mar-2020	BC
Resource Cruise 01 (RC01)	BC_029	UKSR-1	12.3657°N, 116.7521°W	4159	10-Mar-2020	BC
5A	BC_337	NORI-D	10.8450°N, 116.1520°W	4131	04-Nov-2020	BC
5A	BC_349	NORI-D	10.3289°N, 117.1972°W	4283	09-Nov-2020	BC
5A	BC_354	NORI-D	10.3159°N, 117.5140°W	4383	10-Nov-2020	BC
5A	BC_355	NORI-D	10.3559°N, 117.1690°W	4278	11-Nov-2020	BC
5A	BC_359	NORI-D	10.5313°N, 117.3040°W	4312	13-Nov-2020	BC
5A	BC_366	NORI-D	10.3984°N, 117.2723°W	4328	16-Nov-2020	BC
5A	BC_370	NORI-D	10.3789°N, 117.1535°W	4305	17-Nov-2020	BC
5A	BC_377	NORI-D	10.3361°N, 117.2050°W	4284	22-Nov-2020	BC
5A	BC_379	NORI-D	10.3549°N, 117.2208°W	4273	23-Nov-2020	BC
5D	BC_386	NORI-D	10.3675°N, 117.1544°W	4305	11-May-2021	BC
5D	BC_389	NORI-D	10.3244°N, 117.1875°W	4280	12-May-2021	BC
5D	BC_391	NORI-D	10.3218°N, 117.1975°W	4280	13-May-2021	BC
5D	BC_393	NORI-D	10.3784°N, 117.1441°W	4309	14-May-2021	BC
5D	BC_395	NORI-D	10.3773°N, 117.1558°W	4302	14-May-2021	BC
5D	BC_399	NORI-D	10.3546°N, 117.1532°W	4290	16-May-2021	BC
5D	BC_402	NORI-D	10.3481°N, 117.1707°W	4282	17-May-2021	BC
5D	BC_407	NORI-D	10.3590°N, 117.2405°W	4298	19-May-2021	BC
5D	BC_412	NORI-D	10.8397°N, 116.1504°W	4132	25-May-2021	BC
5D	BC_415	NORI-D	10.3315°N, 117.1872°W	4284	28-May-2021	BC
5D	BC_416	NORI-D	10.3341°N, 117.1850°W	4285	29-May-2021	BC
5D	BC_419	NORI-D	10.3333°N, 117.1920°W	4287	31-May-2021	BC
5D	MC_140	NORI-D	11.6040°N, 118.0500°W	4368	31-May-2021	MC
7A	BC_427	NORI-D	10.3325°N, 117.1874°W	4281	24-Aug-2022	BC
7A	BC_430	NORI-D	10.3277°N, 117.1840°W	4284	25-Aug-2022	BC
7A	BC_431	NORI-D	10.3303°N, 117.1750°W	4285	26-Aug-2022	BC
7A	BC_435	NORI-D	10.3342°N, 117.1772°W	4284	27-Aug-2022	BC
7A	BC_448	NORI-D	10.3493°N, 117.1690°W	4275	09-Sep-2022	BC
7A	BC_449	NORI-D	10.3539°N, 117.1713°W	4273	09-Sep-2022	BC
7A	BC_451	NORI-D	10.3575°N, 117.1700°W	4273	09-Sep-2022	BC
7B	BC_460	NORI-D	10.3509°N, 117.2485°W	4293	19-Nov-2022	BC
7B	BC_463	NORI-D	10.3343°N, 117.1771°W	4282	21-Nov-2022	BC
7B	BC_465	NORI-D	10.3341°N, 117.1772°W	4282	22-Nov-2022	BC
7B	BC_466	NORI-D	10.3342°N, 117.1771°W	4282	22-Nov-2022	BC
7B	BC_468	NORI-D	10.3537°N, 117.1714°W	4250	26-Nov-2022	BC
7B	BC_470	NORI-D	10.3552°N, 117.1687°W	4275	27-Nov-2022	BC
7B	BC_471	NORI-D	10.3573°N, 117.1698°W	4273	27-Nov-2022	BC
7B	BC_476	NORI-D	10.3250°N, 117.1810°W	4287	30-Nov-2022	BC
7B	BC_478	NORI-D	10.3336°N, 117.1860°W	4279	02-Dec-2022	BC
7B	BC_481	NORI-D	10.3340°N, 117.1859°W	4279	03-Dec-2022	BC
8A	BC_490	NORI-D	10.3597°N, 117.2445°W	4290	25-Nov-2023	BC
8A	BC_501	NORI-D	10.3541°N, 117.2424°W	4290	13-Dec-2023	BC
8A	BC_502	NORI-D	10.3549°N, 117.2459°W	4289	14-Dec-2023	BC

The material studied was collected during nine expeditions to four different exploration contract areas (henceforth, contract areas) (UKSR-1 [UK Seabed Resources Ltd, United Kingdom], OMS [Ocean Metals Singapore], BGR [Bundesanstalt für Geowissenschaften und Rohstoffe], NORI-D [Nauru Ocean Resources Inc.]) in the CCZ. The ABYSSLINE-2 (ABYSSal baseLINE project; Smith et al. 2015) expedition, on board the R/V “*Thompson*”, was conducted in 2015 and collected samples from the UKSR-1 and OMS contract areas. This expedition also sampled an Area of Particular Environmental Interest (APEI 6). Five cruises to the NORI-D contract area (C5A in 2020; C5D in 2021; C7A and C7B in 2022; and 8A in 2023) collected samples following methods in Glover et al. (2015), and three expeditions collected material from the BGR contract area on board the R/V “*Kilo Moana*” (MANGAN 2016 [Rühlemann et al. 2017] and 2023 [Rühlemann et al. 2023]) and R/V “*Sonne*” (MANGAN 2018; Rühlemann 2018 (Table 1).

The material was collected using either an epibenthic sledge (EBS) or an USNEL spade box corer (BC). Samples from the ABYSSLINE and MANGAN cruises were collected using a Brenke-type epibenthic sledge (EBS, Brandt and Barthel 1995; Brenke 2005). The deployment protocol followed Brenke (2005). Upon recovery, samples were sieved using a 300 µm sieve and either sorted immediately and preserved in 80% ethanol kept at −20 °C or immediately transferred into chilled (−20 °C) 96% ethanol, with sorting carried out after 48 h storage in a −20 °C freezer (Riehl et al. 2014). Samples from NORI-D cruises were collected using an USNEL spade box corer (Ocean Instruments BX-650) with a sample dimension of 50 × 50 cm. Post-collection processing followed Glover et al. (2015). Briefly, surface water is removed and sieved using a 300 µm sieve. Sediment surface biota and nodules were treated separately. A “live-sort” subsample of 15 × 15 cm^2^ of sediment (sliced at 0–2 cm and 2–5 cm) was sieved using a 300 µm sieve, then individual animals were picked, identified, photographed, and preserved individually in 80% ethanol for high-quality DNA and morphology. All remaining sediment was sliced in 0–2 cm, 2–5 cm and 5–10 cm layers, sieved using 300 µm sieves and bulk fixed in 100% ethanol. All specimens (surface water, surface biota, live sort and bulk-fixed sediment) and data are merged for later analyses.

For both collecting methods, selected specimens were subjected to cytochrome *c* oxidase subunit I gene (COI) barcoding and were morphologically identified as operational taxonomic units (OTU). Taxa that were deemed suitable for description were targeted for taxonomic description during the workshop organized at the University of Lodz funded by the International Seabed Authority’s Sustainable Seabed Knowledge Initiative: One Thousand Reasons campaign.

### DNA extraction, amplification, and sequencing

Barcoding of the whole material collected during ABYSSLINE-2, MANGAN 2016, 2018, and 2023 was a goal of another study and the molecular methods used to provide barcodes for species described in this Special Issue are described by Jażdżewska et al. (2025).

Specimens collected from the NORI-D contract areas were extracted and sequenced as detailed in the individual papers using those specimens (Horton and Lörz 2026 Horton et al. [2026a, b, c, d]).

All sequences are deposited in GenBank with the accession numbers as reported in the individual papers. For specimens collected during ABYSSLINE-2 and MANGAN cruises, voucher information, taxonomic classifications and sequences are deposited in the data set “DS-AMPHICCZ” in the Barcode of Life Data System (BOLD) (https://doi.org/10.5883/DS-AMPHICCZ; https://www.boldsystems.org) (Ratnasingham and Hebert 2007) and Barcode Index Numbers (BINs) were ascribed to all of them (Ratnasingham and Hebert 2013).

### Workshop description process

**Figure 2.**
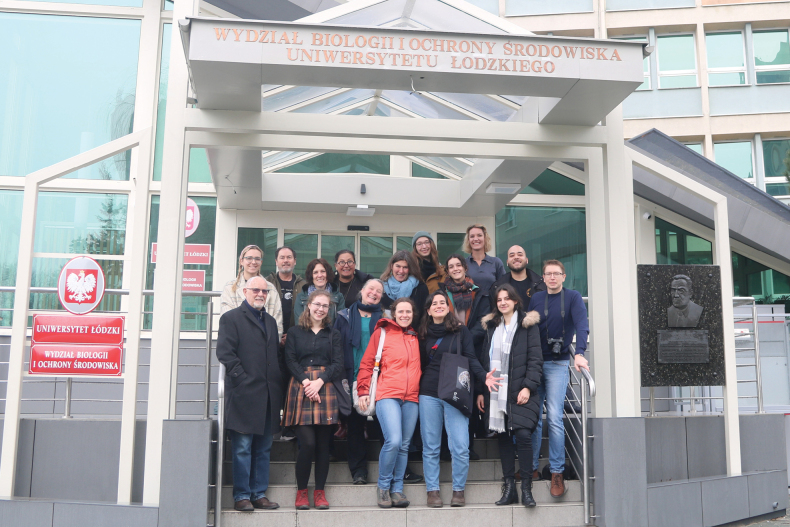
Attendees at the SSKI taxonomy workshop at the University of Lodz (back row left to right: Eva Stewart, Ed Hendrycks, Tammy Horton, Rachael Peart, Anne-Nina Lörz, Laura Engel, Halina Jereczek, Lauren Hughes, Luiz Felipe Anrade. Front row left to right: Krzysztof Jażdżewski, Roxana Timm, Anne Helene S. Tandberg, Anna Jażdżewska, Georgina Valls Domedel, Karolina Biniek, Tomasz Mamos).

In February 2024, in the Department of Invertebrate Zoology and Hydrobiology, University of Lodz, an amphipod taxonomic workshop was organised to facilitate the description of as many new species as possible from the available material. This was possible thanks to a grant entitled “New species of Amphipoda from Clarion-Clipperton Zone” from the International Seabed Authority’s Sustainable Seabed Knowledge Initiative (One Thousand Reasons programme). During the 10-day workshop, a group of 16 participants (eight taxonomic experts and eight early career taxonomists; Fig. 2) began the description process for the new taxa.

Prior to the workshop, extensive work had already been undertaken by the authors of this manuscript to delimit the species to be targeted using both morphological and molecular methods. This initial species delimitation, barcoding and slide preparation allowed the work during the workshop to proceed at pace with the illustration of already defined new taxa during the 10-day process. The habitus of selected species to be described were obtained with a confocal laser scanning microscope (CLSM). In these cases the specimen was stained in Congo red and acid fuchsin, temporarily mounted onto slides with glycerol and examined with a Leica TCS SPV equipped with a Leica DM5000 B upright microscope and three visible-light lasers (DPSS 10 mW 561 nm; HeNe 10 mW 633 nm; Ar 100 mW 458, 476, 488 and 514 nm), combined with LAS AF 2.2.1 software (Leica Application Suite, Advanced Fluorescence). A series of photographic stacks was obtained, collecting overlapping optical sections throughout the whole preparation (Michels and Büntzow 2010; Kamanli et al. 2017).

Specimens were dissected and mounted on slides and illustrations were made according to the methods in each of the submitted papers. The description work was continued by the individual participants, and the final outputs of this workshop are now presented here in this Special Issue.

## Results and discussion

The 24 new species (one new superfamily, one new family, two new genera) are listed in Table 2 along with the contract areas in which they have been recorded. The described species are from 10 amphipod families: Ampeliscidae (2), Eusiridae (5), Lepechinellidae (2), Liljeborgiidae (2), Pardaliscidae (2), Phoxocephalidae (2), Stilipedidae (2), Synopiidae (2), and Tryphosidae (2), and the new family Mirabestiidae (1).

**Table 2.** Taxa described in this Special Issue with their taxonomic position, source regions (UKSR-1: UK Seabed Resources Ltd, United Kingdom, OMS: Ocean Metals Singapore, BGR: Bundesanstalt für Geowissenschaften und Rohstoffe, NORI-D: Nauru Ocean Resources Inc., APEI 6: Area of Particular Environmental Interest) and number of individuals collected. Note: this publication is not deemed to be valid for taxonomic/nomenclatural purposes (see Article 8.2 in the International Code of Zoological Nomenclature 4^th^ Edition [International Commission on Zoological Nomenclature 1999]).

	Superfamily	Family	Species	Authority	Area	No of ind.
1.	Iphimedioidea	Stilipedidae	*Alexandrella haubeni* sp. nov.	Tandberg & Hughes, 2026	BGR	1
2.			*Astyra mclaughlinae* sp. nov.	Tandberg & Hughes, 2026	UKSR-1	1
3.	Eusiroidea	Eusiridae	*Cleonardo compassionate* sp. nov.	Lörz, Engel, Jereczek, Ćwierz & Jażdżewska, 2026	OMS, BGR	4+1*
4.			*Cleonardo daniela* sp. nov.	Lörz, Engel, Jereczek, Ćwierz & Jażdżewska, 2026	UKSR-1, BGR	11
5.			*Dorotea elizae* sp. nov.	Lörz, Engel, Jereczek, Ćwierz & Jażdżewska, 2026	OMS	1
6.			*Rhachotropis clarionclippertoni* sp. nov.	Lörz, Engel, Jereczek, Ćwierz & Jażdżewska, 2026	BGR	22
7.			*Rhachotropis laure* sp. nov.	Lörz, Engel, Jereczek, Ćwierz & Jażdżewska, 2026	BGR, APEI 6	12
8.	Liljeborgioidea	Liljeborgiidae	*Liljeborgia scylla* sp. nov.	Timm, Stewart, Lörz & Horton, 2026	OMS	1
9.			*Liljeborgia sybilline* sp. nov.	Timm, Stewart, Lörz & Horton, 2026	BGR, NORI-D	5
10.	Haustorioidea	Phoxocephalidae	*Harpinia lobata* sp. nov.	Andrade & Jażdżewska, 2026	UKSR-1	2
11.			*Harpiniopsis pedro* sp. nov.	Andrade & Jażdżewska, 2026	BGR	4
12.	Lysianassoidea	Tryphosidae	*Elimedon breviclunis* sp. nov.	Horton, Valls Domedel Stewart & Hendrycks, 2026b	NORI-D	8
13.			*Elimedon zabka* sp. nov.	Horton, Valls Domedel Stewart & Hendrycks, 2026b	APEI 6	1
14.			*Lepidepecreum myla* sp. nov.	Wróblewski & Jażdżewska, 2026	UKSR-1	1
15.			*Thrombasia ania* sp. nov.	Horton, Valls Domedel & Hendrycks, 2026a	BGR	9
16.	Dexaminoidea	Lepechinellidae	*Lepechinelloides polymetallica* sp. nov.	Peart & Lörz, 2026	OMS	1
17.			*Pseudolepechinella apricity* gen. nov., sp. nov.	Horton & Lörz, 2026	OMS, NORI-D	3
18.		Pardaliscidae	*Eperopeus vermiculatus* sp. nov.	Horton, Valls Domedel, Stewart & Hendrycks, 2026c	BGR, NORI-D	10
19.			*Pardalisca magdalenae* sp. nov.	Biniek, Jażdżewska & Hendrycks, 2026	BGR	1
20.	Synopioidea	Ampeliscidae	*Byblis hortonae* sp. nov.	Peart & Stewart, 2026	UKSR-1, OMS, BGR, NORI-D	16
21.			*Byblisoides jazdzewskae* sp. nov.	Peart & Stewart, 2026	UKSR-1, OMS, BGR	5
22.		Synopiidae	*Austrosyrrhoe hamptonae* sp. nov.	Hughes & Tandberg, 2026	BGR	4
23.			*Syrrhoe manowitzae* sp. nov.	Hughes & Tandberg, 2026	UKSR-1, BGR, NORI-D	6
24.	Mirabestioidea superfam. nov.	Mirabestidae fam. nov.	*Mirabestia maisie* gen. nov., sp. nov.	Horton, Valls Domedel Stewart & Thurston, 2026d	BGR, NORI-D	26

*One individual of this species was collected in the NW Pacific (Jażdżewska and Mamos 2019).

Tandberg and Hughes (2026) describe two new species of the family Stilipedidae, one in the genus *Alexandrella* Chevreux, 1911 and one in *Astyra* Boeck, 1871 bringing the known species to 16 and seven, respectively. *Astyra mclaughlinae* sp. nov. is the first record of the genus in the North-east Pacific, as well as the most tropical and the deepest record. Five new species of the family Eusiridae are described by Lörz et al. (2026). The new species of *Cleonardo* Stebbing, 1888 and *Rhachotropis* S.I. Smith, 1883 genera add to the knowledge of diversity and distribution of these predatory amphipods. Additionally, they add a new species to the little-known genus *Dorotea* Corbari, Frutos & Sorbe, 2019, providing its deepest record. Two new species in the micropredatory genus *Liljeborgia* Spence Bate, 1863 are described by Timm et al. (2026), both of which are unusual in having shallowly cleft telsons (less than 30%). Andrade and Jażdżewska (2026) describe two new species of the family Phoxocephalidae, namely *Harpinia lobata* sp. nov. and *Harpiniopsis pedro* sp. nov.—the former providing the deepest record of the genus, the latter providing the first record of the genus in the abyssal Pacific Ocean. The tryphosid genus *Elimedon* J.L. Barnard, 1962 is revised by Horton et al. (2026b) with the description of two new species doubling the number of species in this genus. Wróblewski and Jażdżewska (2026) describe a new species of *Lepidepecreum* from the CCZ bringing the total in the genus to 39 and providing the first record of the genus at abyssal depths. *Thrombasia ania* sp. nov. described by Horton et al. (2026a) adds the fourth new species from the family Tryphosidae presented in the Special Issue. This new species provides the deepest record as well as the first molecular barcode for the genus. Within the family Lepechinellidae, Horton and Lörz (2026) describe a new genus and species, *Pseudolepechinella apricity* gen. nov. sp. nov., which brings the number of genera in the family to six; and Peart and Lörz (2026) describe the third species, *Lepechinelloides polymetallica* sp. nov., in this rarely recorded genus. Horton et al. (2026c) add the second species, *Eperopeus vermiculatus* sp. nov, to another rare genus, extending the geographic distribution of the genus to the Pacific Ocean. Biniek et al. (2026) provide the description of the large species *Pardalisca magdalanae* sp. nov., which is the deepest record of the genus globally. Two new species are described by Peart and Stewart (2026) within the family Ampeliscidae. *Byblis hortonae* sp. nov is the fifth species in this large genus, which now contains 80 species that inhabit depths greater than 2500 m, while *Byblisoides jazdzewskae* sp. nov. is the tenth species in its genus. Both genera are for the first time recorded in the Clarion-Clipperton Zone. Hughes and Tandberg (2026) describe two species of the family Synopiidae, *Austrosyrrhoe hamptonae* sp. nov. and *Syrrhoe manowitzae* sp. nov.; both species providing the deepest records for their respective genera. Finally, Horton et al. (2026d) describe a remarkable new superfamily, new family, new genus, and new species based on morphological and molecular data. This striking new amphipod is placed in the suborder Senticaudata, but the conical mouthparts are unique within this group. The new species is one of the most common in this habitat and is described from >25 specimens.

Suppl. material 1 lists the amphipod taxa now known from the CCZ region. This list includes a summary of the unnamed taxa extracted from Rabone et al. (2023) as well as the newly described taxa from this Special Issue. We can now recognise 32 amphipod families, 57 genera, and 42 species-level amphipod taxa (excluding pelagic Hyperiidea), which includes records from literature and databases, including some unconfirmed/dubious species records, and taxa retained at genus level or a higher taxonomic level (as stet., indet., or sp. nov. according to Horton et al. 2021). Records such as these provide additional evidence of the amphipod diversity in the CCZ that is yet to be fully identified or described.

This Special Issue provides the outputs of the ISASSKI funded workshop describing amphipods from the CCZ. The resulting manuscripts between them describe 24 new amphipod species, one new superfamily, one new family, and two new genera, and add four additional families, and 15 additional genera to the amphipod taxa known from the CCZ region. This could be considered as having tackled a relatively small number of taxa when compared with the large number of unnamed taxa presented in Rabone et al. (2023) and summarised herein for the Amphipoda. However, the description of these 24 species demonstrate that this is actually a very tractable problem. Here we have tackled the description of 24 species in a single year. Recent study of Amphipoda from CCZ revealed 207 MOTUs with only a few species being widely distributed (Jażdżewska et al. 2025). The majority of these taxa appeared to be singletons or doubletons, and the assemblages differed between eastern and western parts of the CCZ.

How many amphipod species are likely to be found in the CCZ region? While the data in this Special Issue do not allow us to calculate diversity measures, we have summarised what is known thus far of the Amphipoda within the CCZ region using the estimates from Rabone et al. (2023). The data therein were extracted from a variety of sources (including published literature and database records) and consequently remain a very crude estimate. Some of those taxa will be synonyms (including of the species described herein), and it is also recognised that as more areas of the CCZ are sampled and more habitats encountered, diversity estimates will increase (e.g. including seamounts or hills, and more samples from the poorly sampled western CCZ). However, if we assume that the numbers of amphipod species in the CCZ are on the order of around 250 species (Rabone et al. 2023; Jażdżewska et al. 2025), and if continued efforts are made to describe and characterise the fauna using both molecular and morphological methods, ensuring these samples and data are made openly available (as originally proposed by Glover et al. 2018), then within a decade of study (25 species added per year = 250 species) we could be in a position to state that the amphipod fauna of the CCZ is “90% known” rather than “very poorly” known as it is currently generally depicted.

An improved understanding of the fauna of the CCZ is needed to be able to answer questions regarding the potential impacts of deep-sea mining on species in the region. Being able to confidently identify the species found in a defined area (using morphological and/or molecular methods) and subsequently to determine how widely distributed a population is, are critical first steps in our ability to resolve these fundamental questions. The SSKI program is a positive step towards achieving that aim, and the results of this workshop presented in this Special Issue provide a successful example model for continued progress in taxonomic description both within and beyond the Amphipoda.

## Additional information

### Use of AI

No use of AI was reported.

### Author ORCIDs

Anna M. Jażdżewska https://orcid.org/0000-0003-2529-0641

Tammy Horton https://orcid.org/0000-0003-4250-1068

### Data availability

All of the data that support the findings of this study are available in the main text or Supplementary Information.

## Supplementary materials

Supplementary material 1 List of amphipod taxa reported from the CCZ Data type xlsx Explanation note **table SS1**. Including those extracted from Rabone et al. (2023); amphipod taxa in the CCZ Checklist (Rabone et al. 2025) and newly described amphipod species added by this Special Issue. Includes named taxa (species, genera, and families from databases and published literature, pelagic taxa removed). https://binary.pensoft.net/file/1566983This dataset is made available under the Open Database License (http://opendatacommons.org/licenses/odbl/1.0/). The Open Database License (ODbL) is a license agreement intended to allow users to freely share, modify, and use this Dataset while maintaining this same freedom for others, provided that the original source and author(s) are credited. Anna M. Jażdżewska, Tammy Horton
